# Delayed recovery due to exaggerated acid, base and electrolyte imbalance in prolonged laparoscopic repair of diaphragmatic hernia

**DOI:** 10.4103/1658-354X.76477

**Published:** 2011

**Authors:** Rakesh Garg, Jyotsna Punj, Ravindra Pandey, Vanlal Darlong

**Affiliations:** *Department of Anaesthesiology and Intensive Care, All India Institute of Medical Sciences, New Delhi, India*

**Keywords:** *Hypocalcemia*, *hypokalemia*, *laparoscopic surgery*, *metabolic acidosis*, *tissue handling*

## Abstract

The acid, base and electrolyte changes are usually observed in the perioperative settings. We report a case of prolonged laparoscopic repair of left-sided diaphragmatic hernia which involved a lot of tissue handling and fluid replacement leading to acid, base and electrolyte imbalance. A 42-year-old male underwent prolonged laparoscopic repair under general anesthesia. Intraoperatively, surgeon reported that contents of hernia includes bowel along with mesentery, spleen and lot of fatty tissue The blood loss was about 2 L which was replaced with 1 L of colloid and 7.5 L of lactated ringer. Near the end of surgery arterial blood gas analysis revealed metabolic acidosis, hyperkalemia, and hypocalcemia leading to delayed recovery. We conclude prolonged laparoscopic surgery involving lot of tissue handling including gut and fat should be monitored for acid, base, electrolyte imbalance and corrected timely to have uneventful rapid recovery.

## INTRODUCTION

The acid, base and electrolyte changes are usually observed in the perioperative settings. Usually such derangements are related to disease pathology but may also be iatrogenic.[1] Intraoperatively this may be caused by the excessive tissue handling, surgical dissection and also by the nature of fluid administration. This poses a unique challenge to anesthesiologist perioperatively. We report a case of prolonged laparoscopic repair of diaphragmatic hernia which involved a lot of tissue handling and fluid replacement leading to acid, base and electrolyte imbalance.

## CASE REPORT

A 42-year-old male weighing 98 kg was scheduled for laparoscopic repair of left-sided diaphragmatic hernia. On reviewing the history, he had multiple episodes of pain on left side of chest mainly after having food since 8 years. He was a known smoker (20 pack years) but had good effort tolerance. On examination, chest auscultation revealed decreased air entry on the left side (mammary, axillary and infraclavicular area). Hemogram, liver and renal function tests were normal.

Chest X-ray revealed high left diaphragmatic dome with right shift of the mediastinum. Electrocardiogram (ECG) was suggestive of left ventricular hypertrophy (probably artifactual due to right shift of mediastinum). Pulmonary function tests showed FVC - 2.62 L (55% predicted), FEV_1_ 2.24 L (57% predicted), FEV_1_ /FVC 85.2% (107% predicted), FEF_25-75_ 2.9 L (68% predicted).

Patient was advised a fasting of 8 hours and was premedicated with oral ranitidine (150 mg) and diazepam (10 mg) the night before and 1 hour before surgery in the morning with a sip of water. In the operation room, routine monitors (ECG, pulse oximeter, non-invasive blood pressure and capnography) were attached. After preoxygenation, anesthesia was induced with fentanyl (180 μg), thiopentone sodium (450 mg) and succinylcholine (150 mg) along with cricoid pressure. Trachea was intubated with cuffed endotracheal tube size 8.5 mm ID. Peripherally inserted central venous catheter was inserted via right cubital vein. Intrathecal morphine (0.3 mg) was administered using 25 G spinal needle in the left lateral position. Lungs were ventilated on pressure controlled mode of ventilation with inspiratory pressure of 20 mmHg and respiratory rate adjusted between 10-15/minute to maintain eucapnia (end-tidal carbon dioxide, EtCO_2_ 35-40 mmHg). Anesthesia was maintained with isoflurane in oxygen and air (MAC 1). Neuromuscular monitor-guided topups of vecuronium was administered. After creating pneumoperitoneum, surgery was started. Intraoperatively surgeon reported that contents of hernia includes bowel along with mesentery, spleen, lot of fatty tissue and was attempted laparoscopic removal. The attempt of removal of the hernial contents continued for about 5 h. We observed that there was lot of tissue handling involving the fat because of adhesions. Among the contents of hernia, the fat content was in abundance and its removal was found to be difficult by laparoscopy via abdominal route and hence thoracotomy was done and herniated contents were retrieved back to abdominal cavity. The blood loss was about 2 L which was replaced with 1 L of colloid and 7.5 L of lactated ringer (RL) intraoperatively over 8 h of surgical duration. Near the end of surgery, arterial blood gas (ABG) analysis revealed pH 7.26, pO_2_ 96 mmHg, pCO_2_ 47 mmHg, HCO_3_ 21 mmol/L, Base excess - 7.7, sodium 142 meq/L, potassium 5.7 meq/L, ionized calcium 0.58 mmol/L. In view of low calcium and high potassium levels, 10 ml of 10% calcium gluconate was slowly administered. 100 meq of sodium bicarbonate was also administered. After the completion of surgery, residual neuromuscular blockade was reversed with neostigmine (3.5 mg) and glycopyrrolate (0.5 mg). Patient was having jerky respiratory efforts and inadequate tidal volume. Considering inadequate reversal, neostigmine (1.25 mg) and glycopyrrolate (0.2 mg) was repeated. Repeat ABG revealed pH 7.23, pO_2_ 206 mmHg, pCO_2_ 60 mmHg, HCO_3_ 24 mmol/L, base excess - 6.7, sodium 143 meq/L, potassium 5.0 meq/L, ionized calcium 0.68 mmol/L. 10 mL of 10% calcium gluconate and 100 meq of sodium bicarbonate was repeated. Patient respiratory efforts were improved and trachea was extubated. Patient was completely pain free. After about 15 minutes of extubation, the patient became slight drowsy and respiratory efforts appears inadequate. Ventilation was assisted using bag and mask with 100% oxygen. Repeat ABG revealed pH 7.28, pO_2_ 473 mmHg, pCO_2_ 49 mmHg, HCO_3_ 23 mmol/L, base excess –6.7, sodium 137meq/L, potassium 6.7meq/L, ionized calcium 0.75 mmol/L. Repeat 100 meq of sodium bicarbonate and 10 mL of 10% calcium gluconate was administered. A 12-lead ECG was suggestive of hyperkalemia. Patient was shifted to high dependency unit and continuous positive airway pressure (CPAP) mask of 7.5 mmHg was applied. Patient remained pain free. Two hours later ABG revealed pH 7.35, pO_2_ 347 mmHg, pCO_2_ 48 mmHg, HCO_3_ 26 mmol/L, base excess-0.9, Na 140meq/L, K 4.9meq/L, ionized Ca 0.91 mmol/L. CPAP was weaned to 5 mmHg in next 12 hours and 24 hours later to face mask. Repeat ABG was normal. Patient was discharged uneventfully 4 days later.

## DISCUSSION

The perioperative management of a patient with long-standing diaphragmatic hernia scheduled for laparoscopic repair can present a unique challenge for the anesthesiologist, specially when hernial contents includes lot of abdominal tissues. In our patient, the surgery took a long time as the contents of the hernia were adhered to adjoining tissue and retrieving the hernia contents were difficult, though laparoscopic retrieval was attempted before proceeding for the thoracotomy. We encountered unique problems of exaggerated acid, base and electrolyte imbalance. To our knowledge, this is first case report of acid, base and electrolyte (potassium, calcium) imbalance in a surgery involving lot of tissue (bowel, fat) handling during laparoscopic diaphragmatic hernia repair.

Patient with major blood loss requires replacement with either crystalloids or colloids including blood products. Blood products are avoided till allowable blood loss to prevent any blood transfusion-associated complications. Lactated ringer is the choice of fluid for such replacement as was done in our patient as well.[2] Our patient had persistent metabolic acidosis requiring its correction with sodium bicarbonate administration. The metabolic acidosis during surgery could occur due to hypovolemia leading to tissue hypoperfusion and lactic acidosis.[2–4] The infusion of normal saline in large volume has been reported to cause hypercholeremic metabolic acidosis but on the contrary RL has been associated to respiratory acidosis as a result of lactate metabolism.[5]

The prolonged pneumoperitoneum has been reported to lead to metabolic acidosis in animal models which could be another probable cause of acidosis in our patient.[6] This could have been due to decrease perfusion of the gut and in our case, manipulation, stretching of the bowel, mesentery and fats tissue from the narrow opening of the hernia would have further decreased the tissue perfusion leading to metabolic acidosis from release of accumulated lactate at the end of surgery, when pneumoperitoneum was deflated and hernial contents were released. The pneumoperitoneum for long duration has been reported to cause metabolic acidosis. Also the decreased tissue perfusion for longer duration can result in acidosis after the perfusion is maintained. This would be the other probability in our case, as perfusion would have been affected to the contents of hernia involving large amount of mesentery along with bowel and fatty tissue due to pneumoperitoneum and also due to surgical manipulation in stretching the hernia contents.

The surgery involving handling of fatty tissue and tissue trauma during retrieval of the herniated contents may lead to electrolyte imbalance. The tissue trauma leads to release of intracellular potassium ions. During surgery requiring lot of tissue handling, chloride and calcium enter the injured cells, causing serum hypocalcemia and potassium leaves the skeletal muscle, producing hyperkalemia, which can cause dysrhythmias and possible cardiac arrest. Phosphate also leaves the cells and results in hyperphosphatemia. Metabolic acidosis ensues from release of lactic acid and other intracellular contents into the circulation.

Hyperkalemia has been reported intraoperatively after extensive muscle dissection during laminoplasty.[7] Fat necrosis due to excessive tissue handling may lead to hypocalcaemia due to binding of calcium to adipose tissues. Hypocalcemia has been associated with pancreatitis, gastrointestinal disorders and bowel surgeries. When the pancreas is damaged, free fatty acids are generated by the action of pancreatic lipase. There are insoluble calcium salts present in the pancreas and the free fatty acids avidly chelate the salts resulting in calcium deposition in the retroperitoneum. Anion chelation of calcium is seen in high phosphate states (e.g., renal failure, rhabdomyolysis, mesenteric ischemia, oral administration of phosphate-containing enemas), high citrate states (e.g., massive blood transfusion, radio-contrast dyes) and high bicarbonate, lactate, and oxalate levels. In our case, lot of fatty tissue trauma probably led to hypocalcemia and required calcium supplementation. Such imbalance may lead to delayed/ inadequate recovery after anesthesia from neuromuscular blockade.

The metabolism of lactated ringer releases carbon dioxide which adds on the respiratory workload. This will be of more concern in prolonged laparoscopic surgery where carbon dioxide is used for creating pneumoperitoneum. This leads to acid/base imbalance and may be associated with inadequate neuromuscular blockade reversal.

We, therefore conclude prolonged laparoscopic surgery involving lot of tissue handling including gut and fat should be monitored for acid, base, electrolyte imbalance and corrected timely to have uneventful rapid recovery.
